# A commentary on ‘Comparative long-term outcomes of natural orifice specimen extraction surgery and conventional laparoscopic colectomy for left-sided colorectal cancer: a propensity score-matched analysis’

**DOI:** 10.1097/JS9.0000000000001637

**Published:** 2024-05-15

**Authors:** Zechuan Wang, Qiang Hu, Fengru Zhou

**Affiliations:** a Department of Traditional Chinese Medicine, Community Health Service Center of Guali, Town of Xiaoshan; b Department of General Surgery, Tongde Hospital of Zhejiang Province, Hangzhou, People’s Republic of China

*Dear Editor*,

We have read with interest the article published by Xu *et al*.^1^. This study confirms that NOSES colectomy using a Cai tube for left-sided colorectal cancer is a safe and feasible option with better cosmetic results, less pain, faster recovery of gastrointestinal function, and comparable long-term clinical and oncologic outcomes to CL colectomy. This is a very important and meaningful study, as it provides us with a new surgical technique and has a significant guiding effect on clinical practice. Although, we believe it is a very interesting topic, we would like to offer the following points for your consideration.

First, the prognostic nutritional index (PNI) was not provided in the manuscript. Multiple studies have shown that the PNI is an independent risk factor for predicting postoperative complications in colorectal cancer^2^. The PNI describes a patient’s nutritional status, inflammatory response, and immune level, which is related to serum albumin concentration and total peripheral blood lymphocyte count. A study showed that the incidence of postoperative complications in the low PNI group was as high as 88.2%, while the incidence of postoperative complications in the high PNI group was 11.8%^3^. If the patient is at a low albumin level before surgery, postoperative complications such as anastomotic leakage, anastomotic bleeding, intestinal obstruction, abdominal infection, and pulmonary infection will increase. If the preoperative lymphocyte count is abnormal, it reflects a decrease in the body’s immune system, and the corresponding immune system’s recognition ability will also decrease, which may lead to tumor recurrence and metastasis.

Second, in the manuscript, whether there is a history of diabetes has not been discussed. Some studies have confirmed that diabetes is an independent risk factor for infectious complications after rectal cancer surgery^4^. As a risk factor for postoperative complications of rectal cancer, diabetes has many mechanisms, including the following two aspects: on the one hand, due to the disorder of glucose metabolism in diabetes patients, their tissue healing is slow, and their anti-infective ability also decreases, which prolongs the healing time of the anastomotic stoma and reduces the body’s resistance to inflammation, thus easily causing infection and anastomotic leakage. On the other hand, diabetes will cause changes in blood vessels throughout the body, leading to arteriosclerosis, thus reducing the blood supply capacity of the anastomosis. Therefore, patients with diabetes have a higher probability of postoperative complications.

Third, in the manuscript, it was not discussed whether both groups should use Enhanced Recovery After Surgery (ERAS) protocol. An increasing number of studies have confirmed that ERAS protocol can reduce the stress response of patients during surgery, reduce related complications, and thus shorten hospital stay^5^. Currently, ERAS Protocol has been widely used worldwide.

Finally, we hope that the above factors can be further taken into consideration.

## Ethical approval

Not applicable.

## Consent

Not applicable.

## Sources of funding

This study was funded by Science and Technology Planning Project of Traditional Chinese Medicine (No. 2024ZL345),Gastrointestinal surgery of integrated traditional Chinese and Western Medicine (No. 2017-XK-A20), Project of Zhejiang Provincial Department of Education (Y202351378).

## Author contribution

Z.W. and Q.H.: methodology, writing, and review; F.Z.: conceptualization, methodology, writing, original draft preparation, review and editing, and supervision.

## Conflicts of interest disclosure

The authors declare that they have no conflicts of interest.

## Research registration unique identifying number (UIN)

Not applicable.

## Guarantor

Fengru Zhou.

## Data availability statement

This manuscript is a comment. Do not need data availability statement. But all the data during the current study are publicly available.

## Provenance and peer review

My manuscript was not invited.
